# Gait festination in parkinsonism: introduction of two phenotypes

**DOI:** 10.1007/s00415-018-9146-7

**Published:** 2018-12-07

**Authors:** Jorik Nonnekes, Nir Giladi, Anasuya Guha, Urban M. Fietzek, Bastiaan R. Bloem, Evžen Růžička

**Affiliations:** 1 Department of Rehabilitation, Donders Institute for Brain, Cognition and Behaviour, Radboud University Medical Center, PO Box 9101, Nijmegen, 6500 HB The Netherlands; 2 Movement Disorders Unit, Sackler School of Medicine, Sagol School for Neuroscience, Tel-Aviv Medical Centre, Neurological Institute, Tel-Aviv University, Tel Aviv, Israel; 3 Department of Otorhinolaryngology and Head and Neck Surgery, 3rd Faculty of Medicine, University Hospital Kralovske Vinohrady, Charles University, Prague, Czech Republic; 4 Department of Neurology and Clinical Neurophysiology, Schön Klinik München Schwabing, Munich, Germany; 5 Department of Neurology, Donders Institute for Brain, Cognition and Behaviour, Radboud University Medical Center, Nijmegen, The Netherlands; 6 Department of Neurology, Centre of Clinical Neuroscience, First Faculty of Medicine, General University Hospital, Charles University, Prague, Czech Republic

**Keywords:** Festination, Gait, Parkinson’s disease, Freezing of gait, Balance

## Abstract

Gait festination is one of the most characteristic gait disturbances in patients with Parkinson’s disease or atypical parkinsonism. Although festination is common and disabling, it has received little attention in the literature, and different definitions exist. Here, we argue that there are actually two phenotypes of festination. The first phenotype entails a primary locomotion disturbance, due to the so-called sequence effect: a progressive shortening of step length, accompanied by a compensatory increase in cadence. This phenotype strongly relates to freezing of gait with alternating trembling of the leg. The second phenotype results from a postural control problem (forward leaning of the trunk) combined with a balance control deficit (inappropriately small balance-correcting steps). In this viewpoint, we elaborate on the possible pathophysiological substrate of these two phenotypes of festination and discuss their management in daily clinical practice.

**Electronic supplementary material:**

The online version of this article (10.1007/s00415-018-9146-7) contains supplementary material, which is available to authorized users.

## Introduction

Gait festination is among the most characteristic gait disturbances in patients with Parkinson’s disease (PD) or atypical parkinsonism [29]. Festination was already described by James Parkinson in his first essay on ‘The Shaking Palsy’: ‘*The propensity to lean forward becomes invincible, and the patient is thereby forced to step on the toes and fore part of the feet, whilst the upper part of the body is thrown so far forward as to render it difficult to avoid falling on the face. In some cases, when this state of malady is attained, the patient can no longer exercise himself by walking in his unusual manner, but is thrown on the toes and forepart of the feet; being, at the same time, irresistibly impelled to make much quicker and short steps, and thereby to adopt unwillingly a running pace. In some case it is found necessary entirely to substitute running for walking; since otherwise the patient, on proceeding only a very few paces, would inevitably fall*’ [33].

Gait festination is common [19], and often has a disabling impact on the quality of life of affected individuals. In a group of 81 PD patients (mean disease duration 8.5 years), 32% of patients reported to have experienced festination during the previous month [19]. More than half of these patients reported that festination was a disabling problem, and 35% of patients reported frequent falls due to festination. Despite is significance, festination has thus far received relatively little attention in the literature. In this viewpoint, we elaborate on this fascinating phenomenon. Inspired by our joint clinical observations, we first argue that there are two phenotypes of festination, then describe their possible underlying pathophysiological substrate, and finally elaborate on their management.

## Two types of festination

The term festination is derived from the Latin word *festinare* which means *to hasten*. Over the years, several—partly overlapping—definitions of festination have been proposed. Giladi and co-workers described festination as ‘rapid, small steps, done in an attempt to keep the centre of gravity in between the feet while the trunk leans forward involuntarily and shift the centre of gravity forward’ [19]. In contrast, Morris and colleagues shifted the focus to the element of acceleration and progressive shortening of steps: ‘Festination is the shortening of each step in a long gait sequence, together with an increase in gait speed and involuntary forward-leaning of the trunk’ [26].

**Fig. 1:**
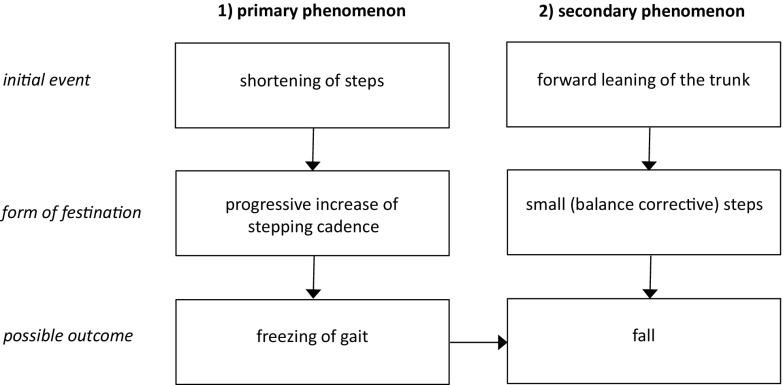
Two phenotypes of festination

Here, we argue that there is not one, but actually two basic phenotypes of festination (Fig. 1). Importantly, we hypothesize that the two phenotypes of festination are not mutually exclusive and can occur in the same patient. The first phenotype entails a primary locomotion disturbance. More specifically, this first basic phenotype of festination is due to the so-called sequence effect: a progressive shortening of step length, accompanied by a compensatory increase in cadence (video 1 and 2). This phenotype most commonly starts in the beginning of walking (e.g. during gait initiation, or after turning). It is strongly associated with freezing of gait: when the progressive shortening of step length and acceleration of step frequency is severe enough, it will ultimately result in gait freezing with alternating trembling of the legs (Fig. 1) [20]. How quickly this happens depends on the ‘baseline’ gait pattern: the smaller the steps, the more likely does freezing occur [9]. Indeed, both festination and freezing are accompanied by rapid movements that tend to occur in the frequency domain of 3–8 Hz [11]. This type of festination frequently coincides with freezing of gait in the same patient. However, it can also occur in isolation, without freezing, if there is no background of severe gait hypokinesia, or if the progressive shortening of steps is not severe enough. Moreover, freezing can also result from a variety of other disturbances in the locomotor network [32], which is another reason why this festination phenotype and freezing do not always coincide. For those patients with another cause for their freezing [16], we hypothesize that this phenotype of festination is not a prerequisite for freezing of gait. Indeed, it is our impression that festination is rare in patients with lesion-induced freezing. However, this should be investigated in future studies.

The second phenotype is not a primary locomotion disturbance, but represents a secondary phenomenon, resulting from a combined postural deficit (forward leaning of the trunk) and a balance control deficit (inappropriately small balance-correcting steps). When there is severe forward leaning of the trunk, the centre of gravity will be located in front of the feet, and the patient will fall forward if no compensatory balance-correcting steps are made [19]. In patients with parkinsonism, these compensatory balance-correcting steps are often too small [10, 27], and are therefore insufficient to restore the centre of gravity within the base of support. Therefore, another balance correcting step is needed to prevent a fall. When this step is again too small, yet another step is needed. Moreover, to prevent falling, patients increase their cadence, and festination emerges (video 3).

This second phenotype can be seen when observing the response to the retropulsion test: the balance-correcting steps are too small to restore the centre of gravity within the base of support, and patients would continue to step backward if they would not be caught by the examiner [28]. This notion raises the question whether a propulsion test (where the patient is pulled towards the examiner standing in front of the patient) is useful to evoke festination in daily clinical practice. A potential drawback is that the distance between the examiner and the patient is not large enough to provoke full-blown festination (unless the examiner rapidly steps backward), but this should be evaluated by future studies. In addition to the retropulsion test, the second festination phenotype can also emerge during gait initiation, or during gait, when the patient makes an unexpected small step, positioning the centre of gravity in front of the base of support [19]. Alternatively, the centre of gravity may also be positioned in front of the base of support due to an external balance perturbation, for example by someone who pushes the patient forward. Finally, forward leaning of the trunk is a dynamic phenomenon that may worsen during walking, resulting in a progressive forward shift of the centre of gravity outside the base of support.

## Festination during other motor tasks

Festination is not restricted to gait, and can also be observed during upper and lower limb tapping tasks [11, 18], or during syllable repetition [24, 25]. We suspect that festination during these tasks is related to the first phenotype of festination, and is thus caused by the sequence effect. Indeed, during these latter tasks, an increase in frequency and decrease in amplitude are typically observed [11, 18].

## Underlying mechanisms

We next elaborate on the possible pathophysiological substrates. The first phenotype of festination is due to progressive shortening steps and acceleration of step frequency. This has been related to defective cue production by the basal ganglia [20]. The basal ganglia are a key component of automatic motor control and are responsible for running each component of a motor plan in a timely manner (motor cue production) [4]. Dysfunction of the basal ganglia in parkinsonism may result in defective cue production, resulting in the above-mentioned sequence effect [9, 20]. Alternatively, dysfunction of the cerebellum might also underlie the first phenotype of festination, as interval timing is not only dependent on the striatum, but also on the cerebellum [6]. An interesting observation in this respect is the fact that cerebellar excitatory theta burst stimulation facilitates gait speed in patients with PD when walking with small steps [22]. Moreover, lesion-induced freezing is associated with lesions within a functional network characterized by connectivity to the cerebellum [16]. Although this is only indirect evidence, future studies might further investigate the role of the cerebellum in the first phenotype of gait festination.

The second phenotype is the result of combined forward leaning of the trunk and small balance-correcting steps. Forward leaning of the trunk (a stooped posture) is common in parkinsonism, but the degree of anterior flexion of the thoracolumbar spine varies considerably across patients, ranging from a relatively mild stooped posture to pronounced forward bending, which is termed camptocormia [13]. Camptocormia can be defined as an involuntary flexion of the spine of at least 30° at the lumbar fulcrum or 45° at the thoracic fulcrum, which is present during standing or walking and resolves in the supine position [15]. Importantly, camptocormia is not a prerequisite for the second phenotype of festination, as a relatively mild stooped posture with marked balance-correcting steps can be sufficient. Additionally, not every patient with camptocormia has the second phenotype of festination, as it is not present in the absence of underscaled balance correcting steps.

A relatively mild stooped posture is presumably directly related to nigrostriatal degeneration, as it is often one of the first presenting signs in PD. On the other hand, previous studies failed to identify one single pathological process for camptocormia [13]. The pathophysiological substrate underlying small balance-correcting steps in parkinsonism has not been unravelled yet [31], but if it relates in any way on the small-stepped gait (which typically improves with dopaminergic stimulation), then nigrostriatal lesions could be involved here as well.

## Treatment

Because the first phenotype of festination is so closely related to freezing of gait, we recommend applying the management guidelines for freezing of gait here. A recent viewpoint on the management of freezing [30] recommended starting treatment by optimizing dopaminergic therapy, and we recommend doing this also in patients with the first form of festination. In addition, as this phenotype seems to be caused by defective cue production, it usually benefits from cueing strategies. The response to cueing tends to depend on the type of external cueing: spatial (visual) cues usually correct and regulate the scaling and amplitude generation during walking, whereas temporal (auditory) cues facilitate gait timing [34, 36]. The effect of these different cueing modalities should always be evaluated in each individual patient, to see which one is most effective [17]. Ambulatory cueing devices, such as visual cueing using a laser shoe [2], or smart glasses that enable visual cueing using augmented reality are now being developed [23], and should be evaluated when these become available for patients.

For the second phenotype, treatment should primarily target the underlying postural control deficits and balance impairment, and not the locomotion disturbance. A mildly stooped posture usually responds to some degree to treatment with levodopa [3], whereas it is generally perceived that camptocormia is not responsive to levodopa [12]. This is probably explained by the generally longer disease duration in patients with camptocormia compared to those with a milder thoracolumbar flexion. Alternatively, patients with camptocormia might have a more specific parkinsonian subtype. Treatment with botulinum toxin injections, DBS and spinal cord stimulation have been evaluated in patients with camptocormia, with varying results, and there is no evidence to support their use [1, 13, 14, 35]. There is also no strong evidence for postural education or the provision of tactile cues using kinesiotape [7], but we usually tend to evaluate its effect by referring to an experienced PD physiotherapist. Management of small balance-corrective steps is also complex. Some studies reported no effect of dopaminergic medication, whereas others reported small beneficial effects [5, 8, 10, 21].

Importantly, all of these management options are based on personal experience and extrapolation from the field of freezing of gait. Having a clearer definition of festination now opens the possibility for further testing of therapeutic options for festination in better-defined populations.

## Conclusion

In this viewpoint, we have introduced a framework for the presence of two phenotypes of festination. This framework may explain several findings and discrepancies that were observed in previous studies on festination [19, 20]. However, as our framework is based merely on the joint clinical experience of the authors, it now needs to be formally validated by future experimental work.

## Electronic supplementary material

Below is the link to the electronic supplementary material.

Video 1 First phenotype of festination (example 1) in a patient with Parkinson’s disease (MP4 20140 KB)

Video 2 First phenotype of festination (example 3) in a patient with Parkinson’s disease (MP4 7846 KB)

Video 3 Second phenotype of festination in a patient with Parkinson’s disease (WMV 1310 KB)
